# The use of in vitro maturation in stimulated antagonist in vitro fertilization cycles of normo-hyperresponder women due to arrested follicular development: A rescue procedure

**DOI:** 10.4274/tjod.22804

**Published:** 2018-09-03

**Authors:** Şafak Hatırnaz, Süleyman Akarsu, Ebru Saynur Hatırnaz, Ahmet Zeki Işık, Michael Haim Dahan

**Affiliations:** 1 Medicana International Hospital, In Vitro Fertilization Center, Clinic of Obstetrics and Gynecology, Samsun, Turkey; 2 Yüksek İhtisas University Faculty of Medicine, Department of Obstetrics and Gynecology, Ankara, Turkey; 3 Medicalpark Hospital, In Vitro Fertilization Center, Clinic of Obstetrics and Gynecology, İzmir, Turkey; 4 McGill University Faculty of Medicine, Fertility Center, Department of Obstetrics and Gynecology, Montreal, Quebec, Canada

**Keywords:** Rescue, in vitro maturation, cycle cancellation, in vitro fertilization, pregnancy rate

## Abstract

**Objective:**

To evaluate the impact of rescue in vitro maturation (IVM) on the clinical outcomes of women with arrested follicular development in stimulated in vitro fertilization (IVF) cycles.

**Materials and Methods:**

This is a retrospective review of 13 patients who were evaluated as normo-hyperresponders for ovarian stimulation. The main outcome measure was the clinical pregnancy and livebirth rates. The purpose of gonadotropin stimulation in patients undergoing IVF is to retrieve multiple oocytes by avoiding multifetal gestation and Ovarian Hyperstimulation syndrome (OHSS). The ovarian response to stimulation ranges from poor response to OHSS, which is related to the follicular number and the dose of the gonadotropins used. However, in some cycles of normo-hyperresponder women, follicular development decelerates or ceases. Close follow-up in a daily manner and increasing the dose of gonadotropins did not change the follicular arrest. This clinical situation has two edges; one is cycle cancellation, which has undesired psychological outcomes for women and the IVF team, and second one is the prolongation of the IVF cycle. For such circumstances, IVM may be a valuable option. Stimulated IVF cycles were converted to IVM as a rescue IVM procedure following detailed informed consent of the women who were close to cycle cancellation.

**Results:**

Thirteen 13 IVM cycles and their clinical outcomes are presented. Six women achieved pregnancies, but only 4 delivered 5 healthy live born. The other two women had biochemical loss during follow-up.

**Conclusion:**

Based on the data obtained, it can be concluded that gonadotropin-stimulated cycles with follicular arrest at the edge of cancellation can be shifted to rescue IVM procedures with reasonable clinical outcomes.

**PRECIS:** Rescue in vitro maturation for arrested follicular development in stimulated cycles.

## Introduction

The story of in vitro fertilization (IVF) began with immature oocytes from unstimulated cycles and finally succeeded with the birth of Louise Brown in 1978^(1)^; however, the roots of studies about immature oocytes and in vitro oocyte maturation (IVM) go back to the 1930s^(2)^. Clomiphene citrate (CC) was discovered in the 1960s and following the livebirths after IVF, the first stimulations were made with CC and multifollicular development in IVF cycles were achieved. Later the addition of human menopausal gonadotropins eased the course of IVF and increased the success rates, but also increased drug-related life-threatening complications such as Ovarian Hyperstimulation syndrome (OHSS). Tremendous use of gonadotropins resulted in the advent of recombinant drugs used in IVF practice. In late the 1990s and early 2000s, the discussion was around reverting to natural or semi-natural cycle managements^(3,4,5)^. In the 1990s, IVM babies were born and IVM gained attention in assisted reproductive technologies (ART)^(6,7)^. To date, the total number of babies born from IVM is around 5000, which cannot be compared to the huge number of babies born from conventional IVF (>7 million). This means that IVM carries some controversies and is not a first-choice treatment in ART^(8)^. The most susceptible women for IVM treatment are patients with Polycystic Ovarian syndrome (PCOS); the best clinical outcomes in IVM cycles were obtained in patients with PCOS^(8)^. The second most common reason for using IVM is to avoid OHSS because patients with PCOS are vulnerable to OHSS^(9)^. IVM has been studied extensively in women PCOS, but indications other than PCOS such as normoresponder women, poor responder women, fertility preservation, rescue IVM for preventing OHSS in stimulated cycles^(10)^, oocyte maturation problems, and patients with cancer who need urgent fertility preservation were introduced into IVM practice^(11,12,13,14,15)^. This study is the first to present IVM shifted from the conventional stimulated IVF cycles due to arrested follicular growth in order to rescue cycles from cancellation. Thirteen normo-hyperresponder patients whose gonadotropin antagonist stimulation cycles were shifted to IVM with human chorionic gonadotropin (hCG) priming were evaluated. All follicles arrested 12 mm and less in size and antagonist drugs were not used in any of the patients. Among the 13 patients, 6 pregnancies were achieved, 2 pregnancies were lost as biochemical pregnancies and the remaining 4 patients delivered five babies with good health. The clinical outcomes of this rescue IVM is acceptable and promising.

## Materials and Methods

This is a retrospective case series of 13 women who underwent rescue IVM therapy at Clinart IVF Center, a private center in the Trabzon province of Turkey, between May 2011 and September 2014. Detailed informed consents of the patients were signed and registered. Institutional Review Board Clinart International Hospital approval by a grant number of 000280/18.05.2015 is present for this trial. For the normo and hyper-responder patients included in the study, a gonadotropin-antagonist protocol was the preferred treatment for ovarian stimulation. The drugs used were: Gonal-F flacon 150-300 IU subcutaneous (sc) once daily (recombinant follitropin alfa, Merck Serono, Switzerland) as recombinant follicle stimulating hormone (FSH) and Cetrotide 0.25 mg sc once daily (cetrorelix acetate 0.25 mg for sc injection, Merck Serono, Switzerland) as gonadotropin-releasing hormone (GnRH) antagonist. Women whose husbands had severe oligoasthenoteratospermia, azoospermia, or cryptozoospermia were excluded. Each patient underwent ovarian stimulation therapy for 5 to 6 days, in regards to mentioned protocol. However, GnRH antagonists could not be administered because of arrested follicular response. Patients with inadequate follicular growth or follicular arrest were given detailed information about the rescue IVM treatment as a valuable option, instead of cycle cancellation. In patients who approved rescue IVM therapy, oocyte retrieval was performed 36 hours after hCG priming with 10.000-20.000 IU/IM. The length of the cycles was similar to FSH priming IVM but shorter than stimulated cycles. Oocyte retrievals of the patients were performed via a 16-gauge double-lumen aspiration needle (Swemed by Vitrolife, Sweden) with low-pressure continuous flushing and collected in a heparinized collection medium. There are no established criteria to identify the ideal timing or method for oocyte retrieval, with most studies using a lead-follicular diameter of up to 12 mm. Lead-follicle diameters greater than 13 mm have been associated with reduced numbers of collected and matured oocytes, possibly related to subsequent atresia of the non-dominant follicles from withdrawal of endogenous FSH support. The aspiration technique for immature oocytes also differs compared with conventional IVF. Transvaginal ultrasound-guided oocyte collection was performed with an aspiration pressure of 100 mmHg. Although the pressure is usually set between 50 and 80 mmHg, in this protocol the pressure was increased to a maximum 100 mmHg instead of prolonging the time of pick-up with low pressure. Extremely high aspiration pressure has been shown to have a negative impact on oocyte development. It takes approximately 10 minutes for oocyte pick-up, and 10 minutes for the evaluation of oocytes per patient. No complications were reported in the oocyte pick-up procedure and all patients were discharged on the same day. The spermatozoa for intracytoplasmic sperm injection (ICSI) were prepared using a three-layer PureSperm gradient (Codes PSB-100 and PS-100-100, Mölndal, Gothenburg, Sweden). The whole medium used for ICSI was prepared and incubated for one day prior to the procedure. Ten milliliters of flushing medium without heparin, and 10 mL of paraffin in Falcon flasks were incubated at 37 °C in atmosphere with high humidity without gas, and a Falcon Center-well dish containing a total of 4 mL of universal IVF medium (Medi-Cult, Code 10311010A) including 1 mL in the center and 3 mL in the perimeter covered with liquid paraffin was incubated at 37 °C in an atmosphere of 6% CO_2_ and 5% O_2_ with high humidity. In addition, a Falcon Petri dish with 40-50 µL droplets of interstellar medium (ISM) 1 medium (Medi-Cult, Code 10500010A) covered with 7 mL liquid paraffin was incubated at 37 °C in an atmosphere of 6% CO_2_ and 5% O_2 _with high humidity. For denudation of the oocytes, 0.7 mL flushing medium without heparin was placed in each well of the four-well dish and covered with liquid paraffin. Eleven microliters of HYASE 100 medium (Vitrolife, Code 10017, 5x0.1 mL, Kungsbacka, Sweden) was added in 1 well of the dish. Removal of the cumulus and corona cells was performed in hyaluronidase-containing medium using Pasteur pipettes after a 26-to-28 hour incubation period. There is no consensus as to which formulation is best suited for the purpose of *in vitro* oocyte culture. The oocytes were then transferred to universal-IVF medium for culture. All ICSI procedures were performed in a Falcon Petri dish with droplets of polyvinylpyrrolidone-containing medium for sperm (Vitrolife, Code 10111, 5x0.1 mL) and droplets of flushing medium without heparin for oocytes. After the ICSI procedure, the oocytes were placed into a ISM 1 medium for culture. In most cases, the fertilized embryos were transferred into the uterine cavity on days 2 or 3. The luteal phase was supported with vaginal progesterone (Progestan 200 mg tablet, Koçak Pharmacy, İstanbul, Turkey) administration once daily and 100 µg transdermal estradiol (Estraderm TTS, Novartis Pharma AG, Basel, Sweden) administration once daily until the fetal heart beat was detected.

### Statistical Analysis

Since this is a case series study without comparisons, we used only excel for analyzing the datas.

## Results

The demographic characteristics, clinical and laboratory parameters are listed in Table 1. The mean age of the women included in the study was 28.3 years with a maximum of 33 and minimum of 24 years. The mean infertility time was 5.4 years. The mean antral follicle count on the third day of the menstrual cycle was 10 in the whole group. Anti-müllerian hormone (AMH) levels were measured in 5 mL venous blood drawn specifically for the present study using enzyme-linked immunosorbent assay. The mean AMH level was 4.79 ng/dL with a maximum of 6.4 and minimum of 2.6 ng/dL. The mean endometrial thickness was 9.9 mm. The mean retrieved oocyte count was 11.15, and the mean retrieval time was 14.4 minutes. The mean number of obtained metaphase II (MII) oocyte was 7.6. Although only one embryo was transferred to 10 of the 13 patients, two embryos were transferred to the remaining 3 patients. Pregnancy was achieved in six patients, two of these six pregnancies were biochemical pregnancies. The remaining four pregnancies resulted in birth of five healthy babies.

## Discussion

The present study shows that IVM converted from stimulated antagonist IVF cycles may be a good alternative approach with favorable outcomes in normo-hyperresponder women whose follicles are resistant to stimulation by gonadotropins. Cycle cancellation due to frustrated follicular growth in normo-hyperresponder patients is an undesirable condition for couples and physicians, and it is not a commonly observed clinical situation. Before cancelling the cycle, day-to-day monitoring or dose oscillations can be used to overcome the follicular arrest. However, in some cases, follicles still resist from growing. Rescue IVM can be offered in such cases to save the cycles from cancellation after giving thorough information about the procedure to couples. It is obvious that, all of the oocytes retrieved in an IVF cycle are not mature, regardless of the chosen IVF protocol. In almost all retrievals, germinal vesicle or MI oocytes were observed, which are discarded from ICSI procedures. IVM has lost its value over time because the clinical outcomes, number of matured oocytes, and developmental competence of embryos are not as good as those obtained from stimulated cycles. Recent advances in ovarian stimulation procedures, safer protocols aimed at decreasing the risk of OHSS, have decreased the attention on IVM^(16,17)^. However, accumulating data from articles supporting IVM because it is safe, cost-effective, simple, repeatable, flexible, and patient friendly in nature, without the risk of OHSS, it serves as a good treatment option. As such, IVF specialists need to be encouraged to add IVM to their clinical practice instead of neglecting it^(18,19,20,21)^. There are few publications regarding rescue IVM in the literature. Jaroudi et al.^(22)^ were the first to report 3 cases in which IVM was used to secure cycles in poor responders, which resulted in deliveries of healthy babies. In a case report from Turkey, Yalcınkaya et al.^(23)^ studied IVM in a poor responder patient who achieved pregnancy and concluded that IVM could be used in poor responders as a good alternative. Braga et al.^(24)^ from Brazil used IVM to mature immature oocytes derived from stimulated cycles but they found that rescue spontaneous maturation of the oocytes did not contribute to clinical pregnancy rates in poor responder women. IVM was studied in patients with repeated oocyte maturation problems from Empty Follicle syndrome (EFS) to oocyte maturation arrest. Patients with Genuine-EFS syndrome (G-EFS) benefited from IVM cycles and achieved ongoing pregnancies^(25)^. In a case report of a woman with a history of repeated G-EFS and azoospermia in her husband, oocytes were retrieved and injected with Mic-TESE derived sperms ; a healthy embryo was transferred but pregnancy was not achieved^(26)^. Another interesting case report revealed that in Resistant Ovary syndrome, IVM worked and oocyte retrieval, embryonic development, and a successful delivery was achieved^(27)^. For managing OHSS, IVM converted from antagonist stimulation cycles were preferred and early hCG priming when the leading follicles were less than 14 mm was planned and favorable laboratory and clinical outcomes were achieved^(11)^. That study resembles our study, but their problem was hyper-response, whereas in our study, the main problem was poor ovarian response in normo-hyperresponsive women. IVM was compared with IVF-ICSI procedures concerning miscarriage rates and Buckett et al.^(28) ^reported that pregnancy loss and clinical miscarriage rates after IVM was higher compared with IVF-ICSI, but this situation was related to PCOS rather than IVM. The oocyte is the central part of folliculogenesis and follicular growth cannot be separated from oocyte development. The oocyte’s journey to maturation is an extraordinary processes and until recently, granulosa cells were thought to be the main contributor of oocyte growth, but the oocyte itself seems to be the key factor in maturation. Two oocyte-derived factors, growth differentiation factor-9 and bone morphogenetic protein-15, moderate regulatory functions and play an important role in oocyte-granulosa cells interaction^(29,30,31)^. This means that any problem in the follicular environment may interfere with oocyte maturation and also follicular maturation. Apoptotic factors found in the follicular fluids of women with G-EFS explain the early oocyte loss in the follicles, thus IVM remains the treatment of choice in patients with G-EFS. FSH and hCG priming alone in IVM cycles are less successful than FSH and hCG priming together in these cycles. Although the results for FSH and hCG priming together are conflicting in IVM, this approach increases the maturation and fertilization rates and developmental competence when compared with other IVM modalities^(32)^. However, Child et al.^(33) ^studied IVM in unstimulated cycles, cycles primed with FSH, and cycles primed with both FSH and hCG in PCOS, and reported similar maturation, fertilization, and cleavage potential in all IVM modalities. IVM treatment shifted from stimulated antagonist IVF cycles seems like FSH and hCG priming IVM. The only difference is the selected dose at the beginning of the cycle. Similarly, favorable pregnancy results with insemination of IVM oocytes from unstimulated cycles were obtained in a study conducted by Söderström-Anttila et al.^(34)^. In hCG-priming-alone IVM cycles, *in vitro*-matured oocytes have more multinucleation and worse clinical outcomes when compared with FSH-priming-alone cycles^(35)^. However, other studies revealed that priming with FSH alone or FSH with hCG priming together had good embryologic and clinical outcomes compared with unstimulated IVM cycles^(36)^. In another article, Fadini et al.^(37) ^showed that FSH priming together with hCG priming in IVM cycles had better clinical outcomes compared with FSH priming or hCG priming alone.

## Conclusion

hCG-priming IVM can be a good option for women experiencing follicular resistance to gonadotropins in antagonist cycles. By this modality, cycles can be rescued from cancellation with favorable clinical outcomes. Nevertheless, information on the safety of IVM with regard to malformation and developmental outcomes cannot be assessed accurately because only a small number of children have been conceived with IVM.

## Figures and Tables

**Table 1:**
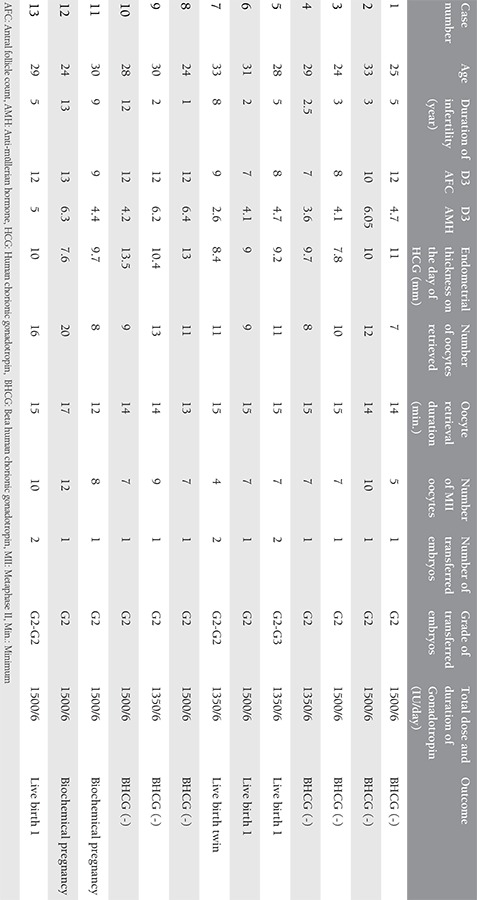
Clinical characteristics of 13 patients
